# Liquid Chromatography-Mass Spectrometry-Based Plasma Metabolomics Study of the Effects of Moxibustion with Seed-Sized Moxa Cone on Hyperlipidemia

**DOI:** 10.1155/2020/1231357

**Published:** 2020-01-22

**Authors:** Qinghua Shao, Jie Cheng, Yi Li, Guangxia Ni

**Affiliations:** ^1^Affiliated Hospital of Integrated Traditional Chinese and Western Medicine, Nanjing University of Chinese Medicine, Nanjing 210028, China; ^2^Jiangsu Province Academy of Traditional Chinese Medicine, Nanjing 210028, China; ^3^College of Acupuncture and Tuina, Nanjing University of Chinese Medicine, Nanjing 210023, China; ^4^Graduate School, Nanjing University of Chinese Medicine, Nanjing 210023, China

## Abstract

Hyperlipidemia (HLP) is a disorder with disturbed lipid metabolism. HLP is a major risk factor in cardiovascular diseases, atherosclerosis, diabetes mellitus, and coronary heart disease. This study focuses on understanding the effects of moxibustion with a seed-sized moxa cone on HLP and the potential metabolic pathways associated with HLP. An automatic analyzer was used to measure the levels of total cholesterol (TC), triglyceride (TG), low-density lipoprotein cholesterol (LDL-C), and high-density lipoprotein cholesterol (HDL-C) in healthy controls (HCs), HLP patients, and in patients before moxibustion with seed-sized moxa cone treatment (BMT) and after moxibustion treatment (AMT). Liquid chromatography-mass spectrometry and pathway analyses were performed using differential plasma metabolites derived from the HC, HLP, BMT, and AMT groups. Higher levels of TC, TG, and LDL-C and lower levels of HDL-C were detected in HLP patients than in HCs. The levels of TC and TG were significantly decreased in the AMT group compared to those of the BMT group. A total of 87 differential metabolites were identified from the HLP vs HC samples and 51 for the AMT vs BMT samples. Of these, 21 terms were shared. The differential metabolites in both the HLP vs HC and AMT vs BMT groups were significantly enriched in the glycerophospholipid and sphingolipid metabolism pathways. We suggest that moxibustion with seed-sized moxa cone treatment is effective against hyperlipidemia by altering the levels of TC and TG, which might be regulated by glycerophospholipid and sphingolipid metabolism.

## 1. Introduction

Hyperlipidemia (HLP) is a disease with disrupted lipid metabolism, and it is associated with high levels of total cholesterol (TC), triglyceride (TG), and low-density lipoprotein cholesterol (LDL-C) as well as low levels of high-density lipoprotein cholesterol (HDL-C) [1]. HLP can increase the risk of many other diseases, including cardiovascular diseases (CVDs) [2], diabetes mellitus [3, 4], periodontitis [3], coronary artery atherosclerosis [5], coronary heart disease [6], and sleep related-breathing disorders [7]. Thus, HLP is a threat to human health and effective therapies are needed.

Moxibustion and acupuncture are potential treatments for HLP [8–10]. The treatment of HLP includes “spleen and stomach, phlegm and dampness.” Chinese medicine considers that the pathogenesis of HLP is related to “phlegm,” which is a major pathologic factor in Chinese medicine [11, 12]. Moxibustion with seed-sized moxa cone has a deeper and more long-lasting stimulatory effect on the acupoints used for the treatment of chronic and refractory diseases, than that of common acupuncture and mild moxibustion treatments. According to the traditional theory and the clinical application of acupuncture and moxibustion, the “fenglong” point (ST40, the national standard code of acupoints) is the first important point for the treatment of “phlegm” and is often used for the treatment of HLP [13]. However, the effect of moxibustion with seed-sized moxa cone on the “fenglong” point of HLP patients and the relative metabolic profiles are still unknown.

Based on liquid chromatography-mass spectrometry (LC-MS) analysis, the plasma metabolic profiles of HLP patients showed 87 differential metabolites for HLP vs HC and 51 for AMT vs BMT. Of these, 21 metabolites were shared. The differential metabolites in both the HLP vs HC and AMT vs BMT groups were significantly enriched in the glycerophospholipid and sphingolipid metabolism pathways.

The present findings might help identify potential metabolic biomarkers and help in developing an effective therapeutic strategy for hyperlipidemia.

## 2. Materials and Methods

### 2.1. Sample Collection

Primary HLP patients (*n* = 23, 10 males and 13 females) and HCs (*n* = 12, 4 males and 8 females) were enrolled. According to the Guidelines for Prevention and Treatment of Dyslipidemia in Chinese Adults (2016 revision), the diagnosis of HLP was confirmed based on one or more of the following criteria: TC > 5.20 mmol/L, TG > 1.7 mmol/L, LDL-C > 3.37 mmol/L, and HDL-C ≤ 1.04 mmol/L. The ages of all participants ranged from 30 to 65 years. If the HLP patients did not take any lipid-lowering drugs or lipid-regulating drugs were being used, they were discontinued for a month. Among 23 HLP patients, 12 were randomly selected to receive moxibustion treatment. The patients were mainly treated by moxibustion with seed-sized moxa cone at the “fenglong” of bilateral calves using approximately 7 to 10 cones at each acupoint, twice a week and continuously for 8 weeks. During the clinical trial, all subjects took regular breaks, followed a reasonable diet, exercised, stopped smoking and consuming alcohol, and avoided drinking strong tea and coffee. Blood samples were collected twice from each person (including HLP patients, HCs, and HLP patients) before and after 8 weeks of treatment. Venous blood samples (6 ml) were collected through the cubital vein at 8 am following an overnight (12 h) fast that began at 8 pm the night before blood collection. Each blood sample was divided into halves. Three milliliters of blood sample was used for evaluation of biochemical parameters, and the other 3 ml was centrifuged at 3000 rpm at 20°C and stored at −80°C. The plasma samples collected before and after moxibustion treatment were designated BMT and AMT, respectively. This study was approved by the ethics committee of the Affiliated Hospital of Integrated Traditional Chinese and Western Medicine (No. 2016LW14). All patients provided written informed consent.

### 2.2. Measurement of Biochemical Parameters

Three milliliters of blood samples was collected in tubes containing anticoagulants from HC, HLP, BMT, and AMT patients after a 12 h fast. The samples were kept at 4°C for 30 min, followed by centrifugation at 3500 rpm for 10 min. The plasma from each sample was packed in a 2 ml centrifuge tube and stored at −20°C until further use. The concentrations of TG, TC, LDL-C, and HDL-C were measured using a model XL-640 automatic analyzer (Erba Diagnostics, Mannheim, Germany).

### 2.3. Sample Preparation for LC-MS

Two hundred microliters of plasma was thawed at room temperature in a 1.5 ml Eppendorf tube. *Cnidium lactonejiae* extract (12.5 *µ*l, 100 *µ*g/ml) was added as the internal standard. After adding 400 *µ*l of ice-cold methanol (75%), the mixture was vortexed for 10 min and then vortexed for 20 min with methyl tertiary butyl ether. After adding 60 *µ*l of ultrapure water, the solution was centrifuged at 12500 rpm for 5 min at 4°C. Following that, 900 *µ*l of the upper layer of the lipid solution was dried in the SpeedVac evaporating air concentrator. After dissolving with methanol/toluene solvent (9 : 1, v : v), the mixture was vortexed at 17500 rpm for 5 min at 4°C. Finally, 600 *µ*l of the liquid supernatant was harvested for LC-MS analysis.

### 2.4. LC-MS

LC analysis was performed using a Kinetex C18 2.6 micron column. Column temperatures were maintained at 55°C. The positive ion sample liquid chromatography used two solutions. Solution A comprised water/isopropanol (80 : 20, v : v) containing 5 mM ammonium formate and 0.1% formic acid. Solution B comprised isopropanol/acetonitrile (90 : 10, v : v) containing 5 mM ammonium formate and 0.1% formic acid. Gradient elution was done using a flow rate of 0.3 ml/min. The elution conditions were 0–4 min, 30% buffer B; 4–5 min, 48% buffer B; 5–22 min, 82% buffer B; 22–23 min, 99% buffer B; and 24–35 min, 15% buffer B. The negative ion sample liquid chromatography also used two solutions. Solution A comprised 0.02% aqueous formate, and solution B was acetonitrile. The flow rate and elution conditions of gradient elution were the same as those of the positive ion chromatography.

MS was performed with electrospray ionization using both positive and negative ion modes. The analytical parameters for MS were as follows: ion source temperature: 350°C; spray voltage: 3.5 kV; sheath gas flow velocity: 275 kPa; capillary temperature: 350°C; and auxiliary air velocity: 104 kPa. The full mass scan range was set to 200 to 1000 *m*/*z* with the resolution ration of 30000 dpi.

### 2.5. Data Preprocessing and Statistical Analysis

The raw data acquired from LC-MS were analyzed using the Progenesis QI software (Waters Corporation, Milford, USA) with parameters such as 5 ppm precursor tolerance, 10 ppm fragment tolerance, and 0.02 min retention time (RT) tolerance. The data profiles included *m*/*z*, peak RT, and peak intensities; RT-*m*/*z* pairs were used as the identifiers for each ion. The combined positive and negative data were imported into the SIMCA software package (version 14.0; Umetrics, Umea, Sweden). Supervised orthogonal partial least squares discriminant analysis (OPLS-DA) was performed to visualize the alterations of metabolites among the groups.

### 2.6. Identification of Differential Metabolites

Metabolites were identified using the Progenesis QI software (Waters Corporation) based on the Human Metabolome Database (HMDB, http://www.hmdb.ca/), LIPID MAPS database (http://www.lipidmaps.org/), and the self-built database of Shanghai Lu-Ming Biotech Co. Ltd (Shanghai, China). The differential metabolites were screened using a combination of multidimensional and unidimensional analysis. The thresholds were set to variable important for the projection (VIP) obtained from the OPLS-DA > 1 and the *P* value from the two-tailed Student's test <0.05.

### 2.7. Pathway Analysis for Differential Metabolites

To identify the effects of the altered metabolites on the metabolic pathways, pathway enrichment analysis for differential metabolites was performed using MBRole 2.0 (http://csbg.cnb.csic.es/mbrole2/) based on Kyoto Encyclopedia of Genes and Genomes (KEGG, http://www.genome.jp/KEGG/pathway.html). The pathways with a *P* value <0.05 were identified as significant pathways.

### 2.8. Statistical Analysis

The experiment data containing the details of the biochemical index are presented as means ± standard error of mean. Statistical analysis of the experiment data was performed using the software SPSS 21.0 (IBM Corp Armonk, NY, USA). A two-tailed Student's *t*-test was used to assess the comparisons between the HC group and HLP group. The paired *t*-test was used to assess the contrast between the BMT group and AMT group. The significance threshold and extremely significance threshold were set at *P* < 0.05(^*∗*^) and *P* < 0.001(^*∗∗*^), respectively.

## 3. Results

### 3.1. Clinical Chemistry Results

**Figure 1:**
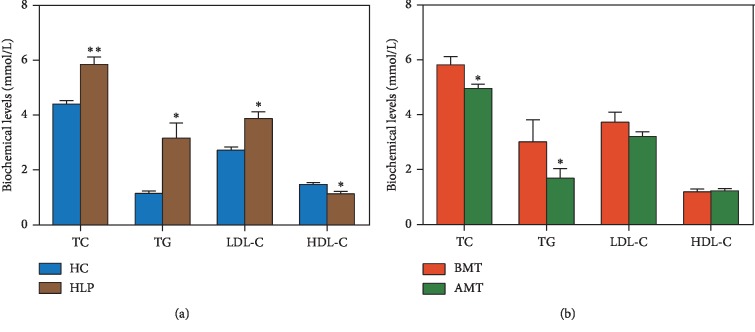
Effects of moxibustion treatment on blood lipid levels in hyperlipidemia (HLP) patients. (a) Levels of total cholesterol (TC), triglyceride (TG), low-density lipoprotein cholesterol (LDL-C), and high-density lipoprotein cholesterol (HDL-C) in HLP patients (*n* = 12) and healthy controls (HC, *n* = 23). (b) Levels of TC, TG, LDL-C, and HDL-C in HLP patients treated with moxibustion (AMT, *n* = 12) and HLP patients without moxibustion treatment (BMT, *n* = 12). ^*∗*^*P* < 0.05; ^*∗∗*^*P* < 0.001.

To explore whether moxibustion with seed-sized moxa cone can treat HLP, the levels of TC, TG, LDL-C, and HDL-C were measured and compared in HLP and HC patients, as well as in BMT and AMT patients. Compared to the HC group, HLP patients had significantly higher levels of TC (*P*=0.005), TG (*P*=0.033), and LDL-C (*P*=0.022) and a markedly lower level of HDL-C (*P*=0.037) (Figure 1(a)). After the 8-week treatment with moxibustion with seed-sized moxa cone, significantly decreased levels of TC (4.96 ± 0.15 mmol/L, *P*=0.039) and TG (1.69 ± 0.35 mmol/L, *P*=0.015) were evident in the AMT group compared with the BMT group (Figure 1(b)). There were no significant differences in the levels of LDL-C and HDL-C between the AMT and BMT groups; the levels of both LDL-C (3.21 ± 0.18 mmol/L < 3.37 mmol/L) and HDL-C (1.23 ± 0.07 mmol/L > 1.04 mmol/L) in the AMT group were within the normal ranges. These results showed that treatment using moxibustion with seed-sized moxa cone was effective in ameliorating HLP, possibly through regulation of the metabolism of lipids, such as TC and TG.

### 3.2. Plasma Metabolomics Analysis

**Figure 2:**
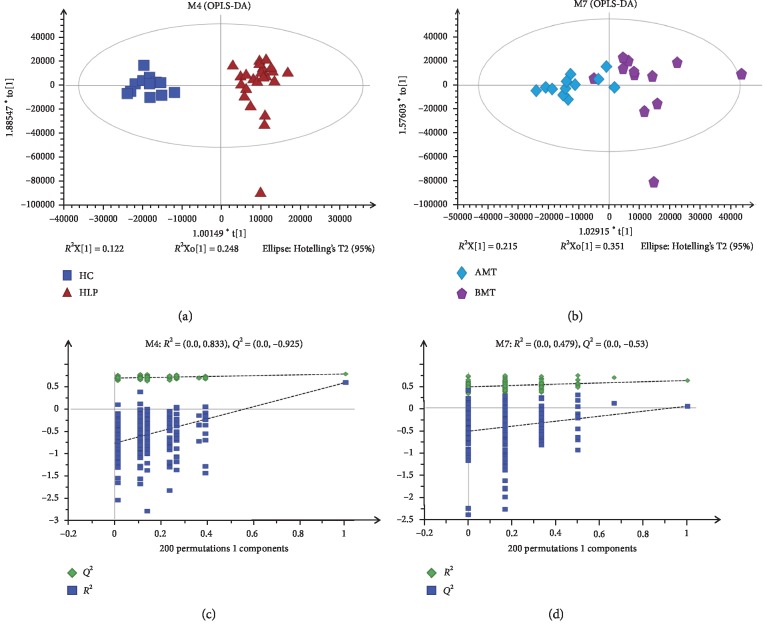
Orthogonal partial lease squares discriminant analysis (OPLS-DA) and permutation testing for plasma samples of HLP, HC, BMT, and BMT. OPLS-DA score plots between HLP and HC (a) and between BMT and AMT (b). Permutation testing of the OPLS-DA models of HLP and HC (c) and OPLS-DA models of BMT and AMT (d).

To evaluate the metabolites in HLP vs HC and BMT vs AMT patients, comparative analysis of the samples was performed using OPLS-DA. The score plots showed a variation between the HLP patients and the HC group (Figure 2(a)) and between the AMT and BMT groups (Figure 2(b)). These results implied that the metabolic profiles were different in the two-pair comparison groups. Permutation testing generated intercepts of *R*^2^ = 0.833 and *Q*^2^ = −0.925 for the OPLS-DA model of HLP and HC (Figure 2(c)) and intercepts of *R*^2^ = 0.479 and *Q*^2^ = −0.53 for the OPLS-DA model of AMT and BMT (Figure 2(d)). These results implied that the OPLS-DA models had a good predictive ability and were not overfitted.

### 3.3. Identification of Differences in Levels of Metabolites

**Figure 3:**
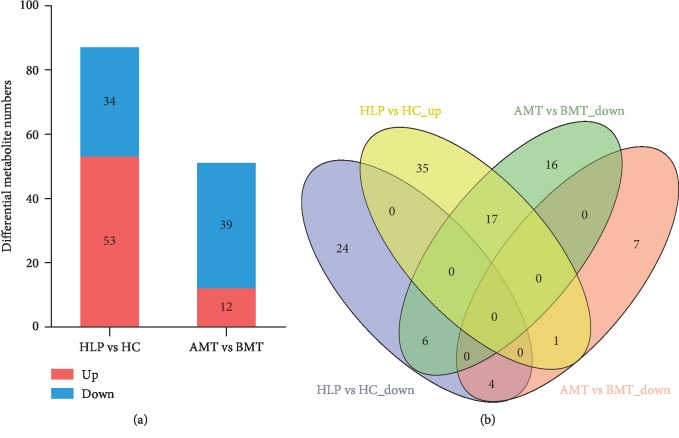
Profiles of differential metabolites in HLP vs HC and BMT vs AMT. (a) Levels of differential metabolites in HLP vs HC and BMT vs AMT; (b) Venn diagram of the differential metabolites common in the HLP vs HC and BMT vs AMT samples.

To further identify the metabolites in HLP compared to HC and BMT compared to AMT, plasma samples from these groups were analyzed by LC-MS. In total, 12,611 metabolites were detected in 47 plasma samples. Compared to HC, 87 metabolites changed markedly in HLP patients, which included 34 metabolites that were decreased and 53 that were increased (VIP > 1, *P* < 0.05) (Figure 3(a)). A total of 51 metabolites were significantly altered between AMT group and BMT group, the levels of 39 were decreased significantly, and the levels of 12 were increased significantly in the AMT group compared to the BMT group (VIP > 1, *P* < 0.05) (Figure 3(a)). Among these differential metabolites, 28 were shared by HLP vs HC and AMT vs BMT (Figure 3(b)). Of these 28 metabolites, 21 had the opposite regulatory correlation, with the levels of 17 metabolites being increased in HLP vs HC, but decreased in AMT vs BMT and the levels of 4 metabolites being decreased in HLP vs HC, but increased in AMT vs BMT. These data revealed that treatment using moxibustion with seed-sized moxa cone might alter metabolite levels in patients with HLP.

### 3.4. Comparison of the 21 Differential Metabolites Shared in the HLP vs HC and BMT vs AMT Groups

**Figure 4:**
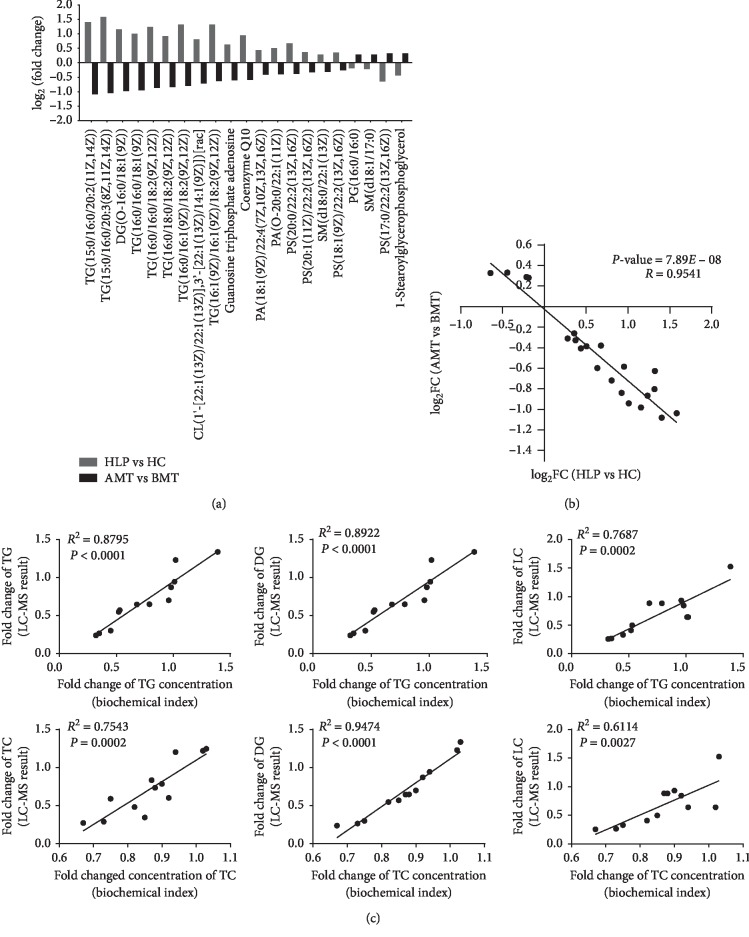
Fold change (FC) of differential metabolites. (a) Change in metabolite levels in the HLP vs HC groups and the BMT vs AMT groups, and linear regression analysis of FC data between the HLP vs HC groups and the BMT vs AMT groups. (b) The black dots denote log_2_ FC of differential metabolites obtained from HLP vs HC (*X*-axis) and BMT vs AMT (*Y*-axis). (c) Correlation between the FC of metabolites and the lowering effect of TC and TG in each patient. The *X*-axis is the FC of TC or TG concentration detected using the XL-640 automatic analyzer. The *Y*-axis is the metabolite changes detected by LC-MS. Each dot is the data from a single patient. R is the correlation coefficient.

To clearly understand the functions of moxibustion with seed-sized moxa cone in the treatment of HLP, we analyzed the 21 shared differential metabolites in the HLP vs HC and BMT vs AMT samples. Of all the metabolites analyzed, the levels of the following were significantly increased in the HLP vs HC groups, but were significantly reduced after moxibustion treatment: CL(1′-[22:1(13Z)/22:1(13Z)], 3′-[22:1(13Z)/14:1(9Z)]) [rac], Coenzyme Q10, DG(O-16:0/18:1(9Z)), guanosine triphosphate adenosine, PA(18:1(9Z)/22:4(7Z, 10Z, 13Z, 16Z)), PA(O-20:0/22:1(11Z)), PS(18:1(9Z)/22:2(13Z, 16Z)), PS(20:0/22:2(13Z, 16Z)), PS(20:1(11Z)/22:2(13Z, 16Z)), SM(d18:0/22:1(13Z)), TG(15:0/16:0/20:2(11Z, 14Z)), TG(15:0/16:0/20:3(8Z, 11Z, 14Z)), TG(16:0/16:0/18:1(9Z)), TG(16:0/16:0/18:2(9Z, 12Z)), TG(16:0/16:1(9Z)/18:2(9Z, 12Z)), TG(16:0/18:0/18:2(9Z, 12Z)), and TG(16:1(9Z)/16:1(9Z)/18:2(9Z, 12Z)) (Figure 4(a)). The 1-stearoylglycerophosphoglycerol, PG (16:0/16:0), PS (17:0/22:2(13Z, 16Z)), and SM (d18:1/17:0) metabolites were significantly decreased in the HLP vs HC groups, but were significantly increased after moxibustion treatment (Figure 4(a)). Additionally, the fold change (FC) of differential metabolites in the AMT vs BMT groups was significantly negatively correlated with that of the HLP vs HC groups (correlation coefficient *R* = −0.9541, *P* < 0.001, Figure 4(b)). To verify the correlation between metabolite changes and the TC- (or TG-) lowering effect in each patient, the correlation of the FCs of metabolites and the lowering effect of TC or TG in each HLP patient treated with moxibustion with seed-sized moxa cone were analyzed. The changes of DG and LC were positively correlated with TC and TG (Figure 4(c)). These data suggested that the metabolite changes induced by moxibustion with seed-sized moxa cone were associated with HLP.

### 3.5. Pathway Analysis of Differential Metabolites

**Figure 5:**
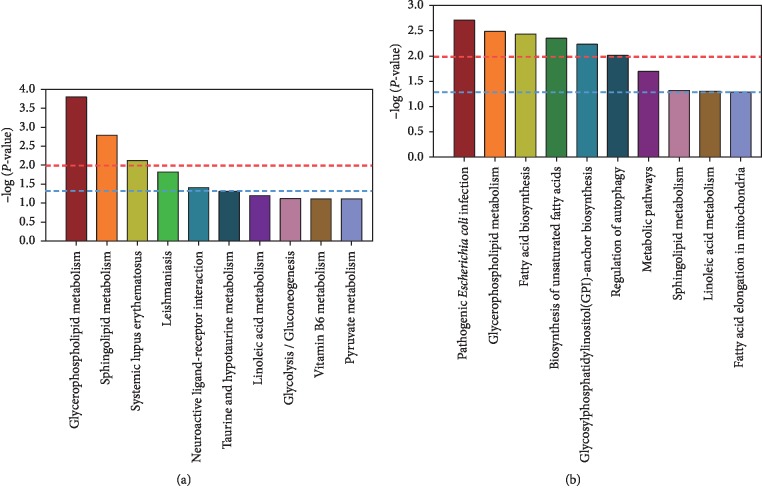
Metabolic pathway analysis of differential metabolites in the HLP vs HC groups (a) and the BMT vs AMT groups (b). The blue and red dotted lines represent *P* < 0.05 and *P* < 0.01, respectively.

To further understand the function of moxibustion with seed-sized moxa cone in the treatment of HLP, the pathways for all the 21 shared differential metabolites were predicted. The top 10 enriched pathways for the differential metabolites of the HLP vs HC groups and the AMT vs BMT groups are shown in Figures 5(a) and 5(b), respectively. The differential metabolites in HLP vs HC that were significantly enriched belonged to the glycerophospholipid metabolism, sphingolipid metabolism, systemic lupus erythematosus, Leishmaniasis, neuroactive ligand-receptor interaction, and taurine and hypotaurine metabolism pathways. The differential metabolites in the AMT vs BMT groups were mainly enriched in pathogenic *Escherichia coli* infections, glycerophospholipid metabolism, fatty acid biosynthesis, unsaturated fatty acids biosynthesis, glycosylphosphatidylinositol (GPI)-anchor biosynthesis, regulation of autophagy, metabolic pathways, sphingolipid metabolism, and linoleic acid metabolism. The glycerophospholipid and sphingolipid metabolism pathways were shared by the differential metabolites in the HLP vs HC groups and the AMT vs BMT groups. The differential metabolites in the AMT vs BMT samples based on the pathways are shown in Table 1. Phosphatidylcholine and phosphatidylethanolamine, which were both decreased after moxibustion treatment, are involved in glycerophospholipid metabolism. The presence of low levels of sphingomyelin in the patients with moxibustion treatment was associated with sphingolipid metabolism. These results suggested that treatment using moxibustion with seed-sized moxa cone could be effective against HLP by altering the levels TC and TG, which in turn might be regulated by the glycerophospholipid and sphingolipid metabolism pathways.

## 4. Discussion

HLP is a widespread disease and a major risk factor in many other diseases. Although many chemical drugs, including statins and fibrates, have been used for the treatment of HLP, most of these drugs are expensive and have negative side effects, such as cognitive disorders, diabetes mellitus, and formation of uterine fibroids [14, 15]. Moxibustion and acupuncture have been considered as potential treatments for HLP [8–10]. We presently conducted an 8-week treatment using moxibustion with seed-sized moxa cone, which resulted in significant downregulation of TC and TG levels. However, there were no significant differences between the AMT and BMT groups. We observed alterations in the levels of 21 metabolites and predicted that the glycerophospholipid and sphingolipid metabolism pathways were involved in HLP and HLP patients treated with moxibustion, respectively. The findings support the conclusion that moxibustion with seed-sized moxa cone is effective against HLP and acts by affecting the TC and TG levels, which might be regulated by glycerophospholipid and sphingolipid metabolism.

We detected higher levels of TC, TG, and LDL-C along with lower levels of HDL-C in HLP patients, than those of the HC patients, which is consistent with the previous literature [16–18]. The 8-week treatment using moxibustion with seed-sized moxa cone significantly downregulated the levels of TC and TG. While there was no significant difference in the levels of LDL-C and HDL-C between the AMT and BMT groups, and the LDL-C and HDL-C levels in the AMT group were both in the normal range. The lack of a significant difference in the levels of LDL-C and HDL-C between the AMT and BMT groups might be due to the insufficient number of patients in the study. The collective results indicate that the moxibustion with seed-sized moxa cone has an anti-HLP effect that involves increased levels of TC and TG.

Moxibustion can cause changes in normal human serum metabolism patterns and enhance the metabolism of amino acids and fatty acids by affecting the concentration of branched amino acids, polyunsaturated fatty acids, and other metabolites [19]. Presently, plasma metabolite profiles were assessed by LC-MS. In total, 21 differential metabolites with opposite correlations were common in the HLP vs HC groups and the AMT vs BMT groups. The significant negative correlations observed between these two groups imply the importance of these metabolites in the progression of HLP. This suggests that moxibustion with seed-sized moxa cone therapy might be beneficial in the treatment of HLP patients.

The differential metabolites in AMT vs BMT were significantly enriched in lipid metabolism pathways (glycerophospholipid metabolism, fatty acid biosynthesis, unsaturated fatty acids biosynthesis, sphingolipid metabolism, and linoleic acid metabolism). The differential metabolites that were common between the HLP vs HC and AMT vs BMT groups involved the glycerophospholipid and sphingolipid metabolism pathways. The associated differential metabolites (phosphatidylcholine, phosphatidylethanolamine, and sphingomyelin) in the moxibustion treated patients might act as potential biomarkers. In recent years, the glycerophospholipid and sphingolipid metabolism pathways have been related to the progression of atherosclerosis, which is accompanied by hyperlipidemia [20]. The enriched metabolites in these two pathways can disrupt lipid metabolism and, hence, play an important role in the occurrence and development of HLP. The concentration of plasma phosphatidylcholine hydroperoxide is significantly increased in HLP patients and have been strongly positively associated with TC and TG levels [21]. Phosphatidylcholine levels are independent risk factors for cardiovascular disease [22–24]. Soybean phosphatidylcholine prevents lipid accumulation and eases high-fat diet-induced hyperlipidemia by decreasing the levels of TC and TG in mice [25]. In cholesterol-fed rabbits, significant increases in the concentration and synthesis rates of sphingomyelin were described [26]. The alterations in the composition of very low-density lipoproteins, which are characterized by a dramatic increase in the levels of sphingomyelin, are similar to the changes in the lipid composition seen in atherosclerosis [27]. One study described that the increased sphingomyelin content in apoE0 mice promoted the occurrence and development of atherosclerosis [28]. Other studies reported that sphingomyelin inhibition can reduce the levels of plasma TC and TG and prevent atherosclerosis in mice [29] and that the levels of TC and TG are independent risk factors for coronary artery disease [30, 31]. In our study, the TC and TG levels were significantly decreased after treatment using moxibustion with seed-sized moxa cone. Thus, the anti-HLP effect of moxibustion likely involved the lowered levels of phosphatidylcholine, sphingomyelin, TC, and TG.

It has been reported that the mean heart rate was decreased after treatment with moxibustion compared with before treatment in patients with qi-deficiency syndrome, which suggests that moxibustion is likely related with activation of autonomic nervous system [32]. The effect of moxibustion may involve the activation of the vagus nerve, as indicated by measurement of heart rate variability in patients with chronic fatigue syndrome [33]. However, in adults with prehypertension, 24 hr systolic and diastolic blood pressures and the 24 hr pulse pressure and pulse rate were not significantly changed before and after treatment with moxibustion in adults. Compared with before treatment, headache, symptoms of flushing, and fatigue were significantly decreased after treatment, suggesting that moxibustion might contribute to alleviating symptoms associated with the autonomic nervous system in adults with prehypertension [34]. Presently, the blood pressure and mean heart rate of 2 patients with primary hypertension before and after each intervention were not significantly changed. Compared with previous studies, we suggest that the treatment using moxibustion with seed-sized moxa cone might contribute to alleviating symptoms associated with the autonomic nervous system, which do not include blood pressure and mean heart rate in patients with HLP. Symptoms, such as headache, flushing, and fatigue, associated with the autonomic nervous system need to be further explored in HLP patients treating using moxibustion with seed-sized moxa cone.

In conclusion, LC-MS revealed the metabolic profiles for HLP, HC, BMT, and AMT patients and identified differential metabolites in comparative analyses of the HLP vs HC groups and the AMT vs BMT groups. The high levels of TC and TG levels found in HLP patients were reversed by treatment using moxibustion with seed-sized moxa cone, indicating anti-HLP effects. Moxibustion with seed-sized moxa cone could regulate lipid metabolism pathways, especially glycerophospholipid and sphingolipid metabolism pathways, to treat HLP. To our knowledge, this is the first study reporting the effects of treatment of HLP using moxibustion with seed-sized moxa cone and the resulting metabolic profile. The optimal treatment conditions with moxibustion, including moxa dosage, treatment time, and treatment cycle, still need to be studied. In addition, further research is required to understand the molecular mechanisms of HLP.

## Figures and Tables

**Table 1:** Pathway terms for the differential metabolites in AMT vs BMT.

Pathway term	*P* value	Metabolites in KEGG	Differential metabolites	FC
Pathogenic *Escherichia coli* infection	1.95*E* − 03	Phosphatidylethanolamine	PE(18:3(6Z, 9Z, 12Z)/P-18:1(11Z))	0.61
			PC(14:0/22:4(7Z, 10Z, 13Z, 16Z))	0.77
			PC(15:0/16:0)	1.12
			PC(18:0/P-18:0)	0.69

Glycerophospholipid metabolism	3.25*E* − 03	Phosphatidylcholine	PC(14:0/24:1(15Z))	0.64
			PC(16:0/P-18:0)	0.18
			PC(14:0/20:2(11Z, 14Z))	0.29
		Phosphatidylethanolamine	PE(18:3(6Z, 9Z, 12Z)/P-18:1(11Z))	0.61

Fatty acid biosynthesis	3.68*E* − 03	Palmitic acid	Palmitic acid	1.17
		Stearic acid	Stearic acid	1.15

Biosynthesis of unsaturated fatty acids	4.45*E* − 03	Palmitic acid	Palmitic acid	1.17
		Stearic acid	Stearic acid	1.15

Glycosylphosphatidylinositol(GPI)-anchor biosynthesis	5.85*E* − 03	Phosphatidylethanolamine	PE(18:3(6Z, 9Z, 12Z)/P-18:1(11Z))	0.61

Regulation of autophagy	9.73*E* − 03	Phosphatidylethanolamine	PE(18:3(6Z, 9Z, 12Z)/P-18:1(11Z))	0.61
		3,4-Dihydroxymandelate	3,4-Dihydroxymandelic acid	0.73
Metabolic pathways		Palmitic acid	Palmitic acid	1.17
		Phosphatidylcholine	PC(14:0/22:4(7Z, 10Z, 13Z, 16Z))	0.77
			PC(15:0/16:0)	1.12
			PC(18:0/P-18:0)	0.69
			PC(14:0/24:1(15Z))	0.64
			PC(16:0/P-18:0)	0.18
			PC(14:0/20:2(11Z, 14Z))	0.29
		Phosphatidylethanolamine	PE(18:3(6Z, 9Z, 12Z)/P-18:1(11Z))	0.61
			TG(16:1(9Z)/16:1(9Z)/18:2(9Z, 12Z))	0.65
			TG(16:0/16:1(9Z)/18:2(9Z, 12Z))	0.57
		Triacylglycerol	TG(16:0/16:0/18:2(9Z, 12Z))	0.55
			TG(16:0/16:0/18:1(9Z))	0.52
			TG(16:0/18:0/18:2(9Z, 12Z))	0.56
			SM(d18:0/18:1(11Z))	0.72
		Sphingomyelin	SM(d18:0/22:1(13Z))	0.81
			SM(d18:0/22:0)	0.59
			SM(d18:0/18:1(11Z))	0.72

Sphingolipid metabolism	4.78*E* − 02	Sphingomyelin	SM(d18:0/22:1(13Z))	0.81
			SM(d18:0/22:0)	0.59
			PC(14:0/22:4(7Z, 10Z, 13Z, 16Z))	0.77
			PC(15:0/16:0)	1.12
			PC(18:0/P-18:0)	0.69

Linoleic acid metabolism	4.97*E* − 02	Phosphatidylcholine	PC(14:0/24:1(15Z))	0.64
			PC(16:0/P-18:0)	0.18
			PC(14:0/20:2(11Z, 14Z))	0.29

Fatty acid elongation in mitochondria	5.16*E* − 02	Palmitic acid	Palmitic acid	1.17

FC: fold change.

## Data Availability

The data used to support the findings of this study are included within the article.
